# Massive parallel sequencing of head and neck conventional squamous cell carcinomas: A comprehensive review

**DOI:** 10.1007/s00428-024-03987-2

**Published:** 2024-11-29

**Authors:** Alfons Nadal, Antonio Cardesa, Abbas Agaimy, Alhadi Almangush, Alessandro Franchi, Henrik Hellquist, Ilmo Leivo, Nina Zidar, Alfio Ferlito

**Affiliations:** 1 Pathology Department, Department of Clinical Fundamentals, Universitat de Barcelona, IDIBAPS, Clínic Barcelona, Barcelona, Spain; 2 Universitat de Barcelona, Barcelona, Spain; 3 Institute of Pathology, University Hospital Erlangen, Friedrich-Alexander University Erlangen-Nürnberg (FAU), Comprehensive Cancer Center (CCC) Erlangen-EMN, Erlangen, Germany; 4 Department of Pathology, University of Helsinki, Helsinki, Finland; 5 Institute of Biomedicine, Pathology, University of Turku, Turku, Finland; 6 Section of Pathology, Department of Translational Research and New Technologies in Medicine and Surgery, University of Pisa, Pisa, Italy; 7 Faculty of Medicine and Biomedical Sciences (FMCB), University of Algarve, Campus de Gambelas, Faro, Portugal; 8 Algarve Biomedical Center Research Institute (ABC-RI), Faro, Portugal; 9 Institute of Biomedicine, Pathology, University of Turku, Turku University Central Hospital, 20521 Turku, Finland; 10 Institute of Pathology, Faculty of Medicine, University of Ljubljana, Ljubljana, Slovenia; 11 International Head and Neck Scientific Group, Padua, Italy

**Keywords:** NGS, Larynx, Oral cavity, Hypopharynx, Oropharynx, Mutation

## Abstract

Head and neck squamous cell carcinoma (HNSCC) is the sixth most common cancer worldwide and is a cause of significant mortality and morbidity. The epidemiology of this cancer varies worldwide due to either genetic differences in populations or differences in carcinogen exposure. The application of massive parallel sequencing-based techniques in HNSCC should provide a helpful understanding of the genetic alterations that eventually lead to HNSCC development and progression, and ideally, could be used for personalized therapy. In this review, the reader will find an overview of the mutational profile of conventional HNSCC according to published results on massive parallel sequencing data that confirm the pivotal role of *TP53* and the frequent involvement of *CDKN2A* and *PIK3CA.* The reader will also find a more detailed description of the genes, such as *NOTCH1* and *FBXW7*, that were not identified in HNSCCs before the development of these techniques, the differences that can be site-specific, such as the different mutational signatures that indicate specific carcinogens for various subsites of the head and neck, and finally, the actionability of these findings that should allow more personalized therapy for patients.

## Introduction

Head and neck squamous cell carcinoma is the sixth most common cancer worldwide and is a cause of significant mortality and morbidity, with populational variations. MPSTs offer insights into genetic alterations in HNSCC and aid in understanding the development and progression of this cancer as well as personalized therapy. In this review, the reader will find an overview of the mutational profile of conventional HNSCC according to published massive parallel sequencing data, a more detailed description of the genes that were not identified in HNSCCs before the development of these techniques, the differences that can be site- or population-specific, and finally, the actionability of these findings. The differences found in non-conventional HNSCCs (HPV-related or representative specific histological subtypes) will be described elsewhere.

**Fig. 1:**
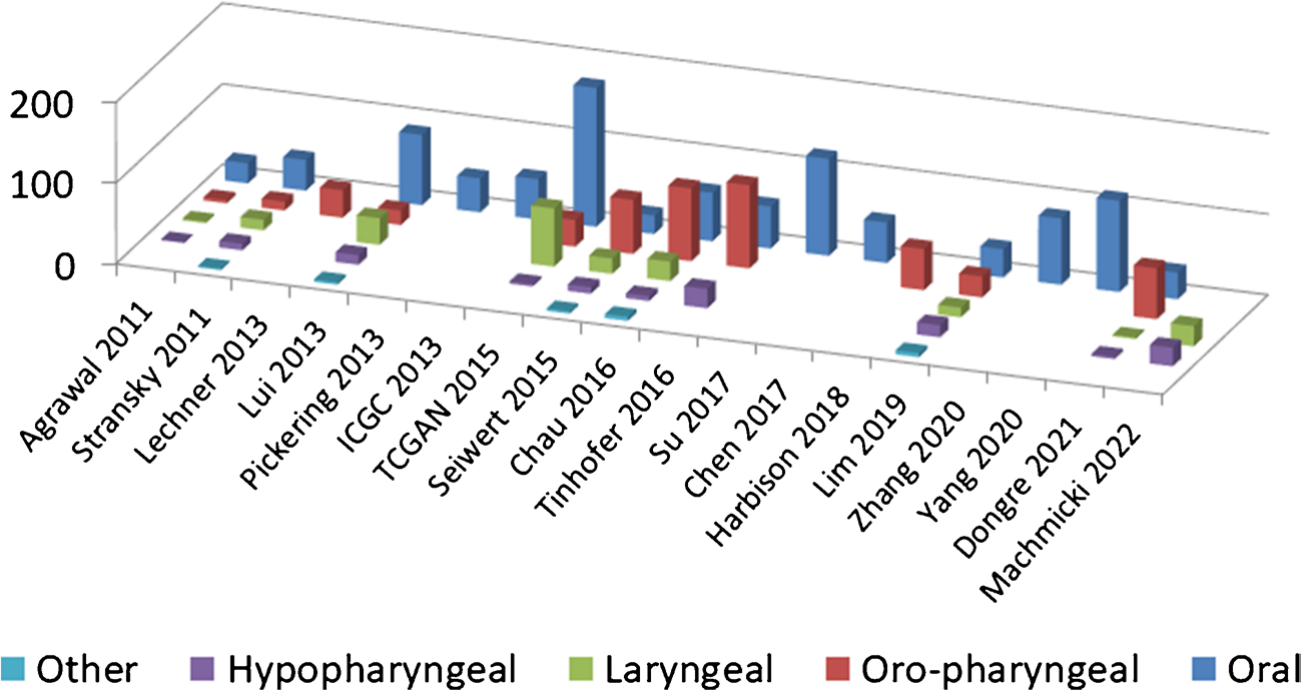
Distribution of absolute numbers of anatomic location of the cases of head and neck squamous cell carcinoma investigated through massive parallel sequencing reported in selected series. References according to the reference list are Agrawal [1]; Stransky [2]; Lechner [21]; Lui [3]; Pickering [10]; ICGC [11]; TCGAN [4]; Seiwert [7]; Chau [8]; Tinhofer [9]; Su [14]; Chen [15]; Harbison [20]; Lim [5]; Zhang [16]; Yang [17]; Dongre [6]; Machmicki [35]

HNSCC occurs in different locations, each with particular etiologies and behaviors. Beginning in 2011, several reports of WES/WGS of HNSCC were published, and although some have attempted to be comprehensive, as they included samples from different anatomic sites, the samples are primarily enriched in oral cavity (OC) (51–78% of the cases), and to a lesser extent, oropharyngeal (OP) (42–61% of the cases) HNSCC [1–9]. Therefore, many reports focus exclusively on OC SCC [10–19] or a combination of OC and OP SCC [20] (Fig. 1).

## Mutation profile of HNSCCs reveals a wide spectrum of unexpected genetic alterations

**Fig. 2:**
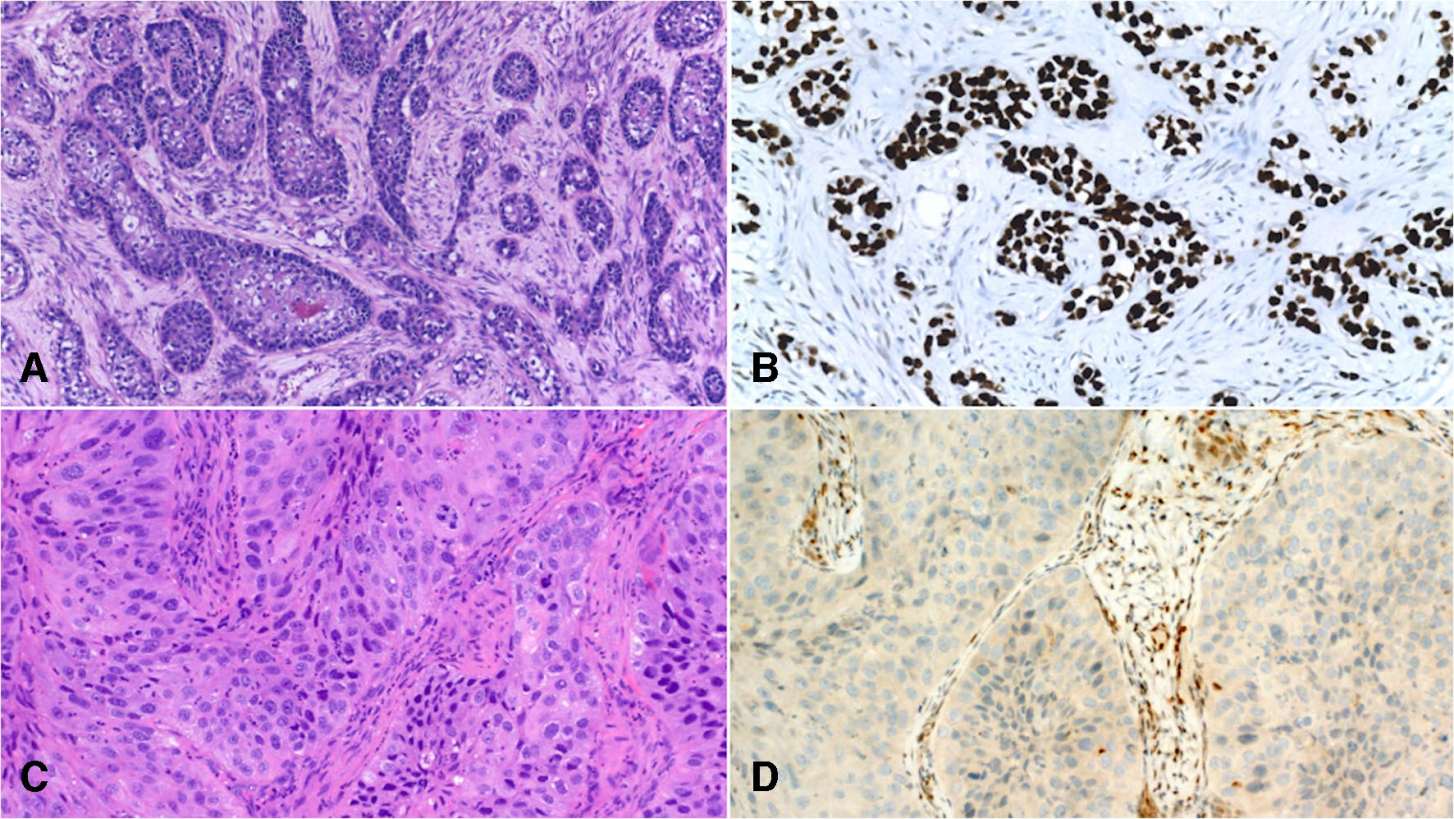
P53 aberrant expression in head and neck SCC. Moderately differentiated SCC (**A**) with diffuse and strong nuclear p53 expression (**B**). Poorly differentiated SCC (**C**) with absent expression of p53 (**D**). Positivity is retained in some stromal cells. Both patterns are indicative of *TP53* mutation

*TP53* mutations constitute the most common mutational event in HNSCCs (Fig. 2) [1, 2, 4–7, 9–11, 14–17, 20, 21], as the prevalence of *TP53* mutations ranges from 43% [6, 14] to 73% [17]. When HPV-related tumors are excluded from the analysis, this range increases to between 66 [5] and 100% [21]. *TP53* mutations are more common among HPV-negative than among HPV-positive tumors [1, 2, 4–7, 9, 20, 21]. In one series, HPV-negative patients harbored fourfold more *TP53* mutations than HPV-positive patients, even though patients in this series exhibited a particularly low prevalence of *TP53* mutations [22].

Mutations in *CDKN2A* are less common, as percentages vary from 22–25% [5] [4] to 9–10% [1, 6]. Inactivation events, in which gene loss or macrodeletions are also considered, occur in 50–58% of cases [1, 4]. *CDKN2A* inactivation events (mutations or gene losses) are more frequent among HPV-negative than among HPV-positive tumors [2, 4–7, 9, 20, 21].

The phosphatidylinositol-4,5-bisphosphate 3-kinase (PI3K) pathway is the most commonly mutated oncogenic pathway, as mutations in this pathway were shown to occur in 30.5% of 152 tumors in one study, which was threefold greater than the percentages of RAS/MAPK- and JAK/STAT-mutated tumors. These tumors have a higher rate of mutations in known cancer genes. All patients whose tumors had concurrent mutations in multiple PIK3 pathway genes were in an advanced (IV) stage, which suggests that concerted PI3K pathway aberrations occur with tumor progression in HNSCC [3]. In another study, approximately one-quarter of the *PIK3CA*-mutated tumors presented with concurrent gene amplification, whereas an additional 20% of the cases presented amplification alone. In all, 73% of *PIK3CA* mutations with known activating effects are located at three hotspots (Glu542Lys, Glu545Lys, and His1047Arg/Leu) [3, 4]. A mutually exclusive pattern of *TP53* and *PIK3CA* mutations has been reported [23].

Previously unreported mutated genes in HNSCC include *NOTCH1* (11%), *NOTCH2* (8%), and *NOTCH3* (11%), which presented loss-of-function mutations in a series of 72 HNSCCs, in contrast to their oncogenic role in lymphoid neoplasms [2]. Mutant *NOTCH1* likely functions as a tumor suppressor gene in HNSCC because *NOTCH1* mutations are frequently combined with LOH or are likely biallelic [1]. Moreover, whereas *NOTCH1* mutations in hematopoietic malignancies are clustered within two hot spots in the heterodimerization (HD) and C-terminal PEST domains, a high fraction of *NOTCH1* mutations in HNSCCs occur in the N-terminal EGF-like ligand binding domain, which alters the protein from the N-terminus to the transmembrane region [1]. *NOTCH1* mutations are prevalent in OSCCs, but in China, some of these variants are localized in the same domains where activating *NOTCH1* mutations are described in hematologic malignancies, which suggest that *NOTCH1* mutations can play opposite roles in different populations [24, 25].

*FBXW7* mutations have not been previously reported in HNSCC [1, 2] [4, 7]. *FBXW7* is a member of the F-box protein family and constitutes a component of the ubiquitin protein ligase complex. This protein acts as a tumor suppressor in several tumors, and one of its major targets is *NOTCH1*. The *FBXW7* mutations observed by Agrawal et al. are in hotspots known to block the degradation of active *NOTCH1* [26]. It is therefore interesting to hypothesize that *FBXW7* mutations modulate the Notch pathway, although *FBXW7* also targets other cancer-related proteins for degradation, including cyclin E and c-myc [1].

Mutations in *CEBPA* (CCAAT/enhancer-binding protein alpha), a tumor suppressor gene mutated in different tumors [27], have not been previously reported in HNSCC, and although the pR323G mutation found in *CEBPA* protein lies in a highly mutated leucine zipper domain, the R323 substitution has not been previously reported [22, 28]. The *FES* (non-receptor protein-tyrosine kinase) pS96L mutation is a new addition to the mutation library of this protein, which has a low mutation rate in TCGAN [22].

Other genes were shown to be frequently altered in HNSCC, including *FAT1* (22%) and *FLG* (19%) in 104 HNSCCs and *MLL2* (17%), *FGFR3* (5–7%), and *HRAS* (4%) in 88 HNSCCs [1] [6] [22], or were shown to be significantly enriched, such as *KMT2D*, *NSD1*, *CASP8*, *AJUBA*, *TGFBR2*, *HLA-A*, *TPRX1*, *CUL3*, *FLG*, *NSD1*, *DDX3X*, *RPIK4*, *KRAS*, *MLL3*, *FGFR2*, *ZNF217*, and *RIMS2* [4] [7, 8]. However, numerous genes, such as *PIK3AP1*, *RIMBP2, SI, NRXN2, NRXN3, EPHA7, RASA1, RXFP3*, *NFE2L2*, *RB1, PTEN, PIK3R1, TRAF3*, *CDH1*, *PIK3R1*, *FLT3*, and *MPL* [1, 4, 22], were altered at lower frequencies. Other mutated genes include those involved in squamous cell differentiation (*IRF6*, *TP63*, *CDH1*, *MLL2*, *MED1, SYNE1, SYNE2, RIMS2,* and *PCLO*), apoptosis (*CASP8* and *DDX3X*) and histone methyltransferase activity (*PRDM9* and *EZH2*) [2].

## Specific sites

Genetic changes in major driver genes are listed in Table 1.

**Table 1:** Major driver pathways

	Major driver pathways	Additional key genes	Specific genes	Specific pathways
Oral cavity	Mitogenic signaling, NOTCH, cell-cycle, and TP53	*FAT1* and *CASP8*	*USP9X, MLL4, ARID2, UNC13C,* and *TRPM3;* recurrent amplifications of *DROSHA* and *YAP1*; homozygous deletions of *DDX3X*; mutations in *GNAQ, PRG4, RP1, ZNF16, BEA*, and *PTPRC* in tongue SCC	Wnt, dorso-ventral axis formation pathway, and axon guidance pathway
Larynx/hypopharyn × (based on Ref. 35)	*TP53*, *FAT1*, *NOTCH1*, *KMT2C (MLL3)*, *CCND1*, and *CDKN2A*	*RTK/RAS/PI3K* and *NOTCH* pathways	*pTERT, CASP8*, and *HRAS* mutations rare or absent	

Mutations in tumor suppressor genes such as *FAT1, CASP8,* and *CDKN2A* are more frequently found in OSCCs than in non-OSCCs, whereas *HRAS* and *PIK3CA* are the only significantly mutated oncogenes [1, 2, 10, 11].

Four major driver pathways (mitogenic signaling, NOTCH, cell cycle, and TP53) plus the additional key genes *FAT1* and *CASP8* were identified in 42 OSCCs. Although only 9% of tumors had *NOTCH1* mutations, the NOTCH pathway was defective in two-thirds of patients. *CASP8* mutations defined a subset of OCs (10% of cases) with fewer copy number changes (5 vs. 19) and demonstrated an association with *HRAS* mutations. Sixty percent of the patients had *TP53* mutations, and 33 of 35 tumors contained alterations in cell cycle pathway genes (*CCND1/CDKN2A*). Twenty-two of 35 tumors presented alterations in genes of the mitogenic signaling pathway, with gains in *EGFR, AKT1, RPS6KB1, PIK3CA,* and *MYC* and activating mutations in *PIK3CA, HRAS,* and *BRAF*. Two-thirds of *FAT1* mutations (seen in 12 of 40 patients) were inactivating mutations and when combined with *FAT1* deletions, as many as 16 of 35 tumors exhibited *FAT1* inactivation [10].

An investigation of genetic heterogeneity in OSCCs revealed that only two identical mutations were found in all tumor samples taken from different areas within a tumor. Similarity was more common between well-differentiated areas than between less differentiated areas within the same tumor. Only *TP53* mutations, but not other driver mutations, were found in multiple sites of any given tumor, which highlights the challenge of OSCC molecular characterization and the importance of tumor heterogeneity in personalized medicine [29].

Some significantly and frequently altered genes are specific for oral and gingivo-buccal SCCs (*USP9X, MLL4, ARID2, UNC13C,* and *TRPM3*), whereas others are shared with other HNSCCs (*TP53, FAT1, CASP8, HRAS,* and *NOTCH1*). Known recurrent cancer genes include *PCLO, FAT3, SMG1, MLL2, SYNE2,* and *EP300*. Pathways enriched for genomic alterations, such as the Wnt signaling pathway, the dorsoventral axis formation pathway, and the axon guidance pathway, have not been previously reported in HNSCC and therefore are considered specific for oral and gingivo-buccal SCCs [11].

Recurrent amplification of the *DROSHA* and *YAP1* genes was reported for the first time, as were homozygous deletions of *DDX3X* in oral and gingivo-buccal SCCs. The *DROSHA* gene, whose gene product is the RNase III protein, is amplified in 12% of 50 OSCC-GB patients, and alterations in the expression of the Drosha gene product have been described in other cancers [11]. *DDX3X* plays an important role in many diseases, including cancer [30].

*GNAQ, PRG4, RP1, ZNF16, BEA,* and *PTPRC* mutations had not been reported until they were found in tongue SCC [16]. *GNAQ*, which encodes a Gα subunit of heterotrimeric G proteins, has been implicated as an oncogene in uveal melanoma [31]. Two of the five missense mutations found in *PRG4* are in the same residue (c.1789A > C), encoding p.(Thr597Pro), which is likely a mini hotspot [16]. *PRG4* has different or even opposite functions in different cells [32] [33], but the functions of *PRG4* and its mutations in tongue SCC have not been elucidated. Mutations in the microtubule-associated protein *RP1* can cause retinitis pigmentosa disorders [34]. *RP1* mutations in tumors were reported for the first time in tongue SCC, while mutations in *PTPRC* and *NBEA* were reported for the first time in solid tumors. Both the *PTPRC* mutation c.1501C > T encoding p.Arg501Trp, which maps to an extracellular domain, and the *NBEA* mutation c.1910 T > C, encoding p.Val637Ala, have been predicted to be highly deleterious. Mutations in *ZNF16* (zinc finger protein 16) were reported for the first time in malignancies, and it was shown that *ZNF16* knockdown significantly diminished cell proliferation, migration, and invasion in vitro and tumor growth in vivo [16].

In laryngeal and hypopharyngeal SCCs, *TP53*, *FAT1*, *NOTCH1*, *KMT2C (MLL3)*, and *CDKN2A* were found to be the most frequently mutated genes. No prominent differences were observed in mutational frequency in nonhypopharyngeal HNSCC, except for the frequency of mutations in the *CASP8* and *HRAS* genes, which ranged from very rare to absent in hypopharyngeal cancers [35].

## Geographic characteristics

Taiwanese patients with OSCC had a high prevalence of mutations in *DHRS4*, *RASA1*, and *SETD8* at rates of 12%, 10%, and 10%, respectively, in series of 50 and 120 cases vs. 1%, 5%, and 1%, respectively, in 279 cases in TCGAN and 0%, 3%, and 0%, respectively, in cases in the ICGC, with *CENVP*, *CHUK* and *ELAVL1* as novel mutated genes [14, 15]. The protein encoded by *CHUK* controls the turnover of cyclin D1 [36, 37]. *CHUK-*mutated tumors exhibit NF-κB activation and *CCND1* overexpression [14]. *ELAVL1* encodes an RNA-binding protein that binds and stabilizes the transcripts of many cancer-related genes [38]. Associated alterations are clustered into two groups: one centered on *TP53, CCND1*, and *CDKN2A* and the other on *HRAS, FAT1, CASP8, EPHA1,* and *NOTCH1* [14].

Novel recurrent mutations in *ASNS* (asparagine synthetase) that may affect substrate binding were found in a series of 6 HPV-negative SCCs in Pakistani patients. The cancer pathways affected included MAP kinase, cell cycle, actin cytoskeleton regulation, and PI3K-Akt signaling [39], and mutations in *ATG2A*, *WEE1*, *DST*, and *TSC2* were identified for the first time in OSCCs [17].

## Tobacco effects

In one study, smoking-related HNSCCs presented nearly universal loss-of-function *TP53* mutations and *CDKN2A* inactivation with frequent CNAs, including amplifications of 3q26/28 and 11q13/22 [4]. The frequencies of *TP53* and *CDKN2A* mutations in OSCCs were similar between smokers and ex-smokers (46% and 42% for *TP53* and 20% and 28% for *CDKN2A* in smokers and ex-smokers, respectively) with similar lifetime tobacco exposure [40].

Smokers harbor 3.2-fold more mutations than nonsmokers [22]. A trend toward a greater number of mutations, amplifications, and driver events was observed in 20 OSCCs from patients with a history of shammah (an Arabian preparation of smokeless tobacco) exposure, particularly those with EBV positivity which suggests an interaction between tobacco exposure and EBV. The results were similar to those reported by the TCGAN: *TP53* and *CDKNA2* mutations (35% of cases for each gene), *CASP8* (30%), *PIK3CA* (20%), *HRAS* (15%), *FAT1*, and *TP63* (10% each); amplifications in *CCND1* (15%) and *FADD (*20%). However, several other candidate novel driver events were also described, including mutations in *NOTCH3* and *CLTCL1* in 15% of cases, mutations in *CSMD3* in 25%, mutations in *CRB1* in 20%, and mutations in *OSMR* and *TRPM2* (10% each); amplification of the proto-oncogenes *FOSL1* (15%), *RELA* (10%), *TRAF6* (190%), *MDM2* (5%), *FRS2* (5%), and *BAG1* (5%); and deletion of the recently described tumor suppressor *SMARCC1* (10%). An analysis also revealed significantly altered pathways not previously implicated in OSCC, including the oncostatin-M signaling pathway, AP-1 and C-MYB transcription networks, and endocytosis. This series was highly enriched in shammah-exposed cases, which could explain why only a trend was detected [13].

*WEE1* and *ATG2A* were mutated at significantly higher rates in 89 areca nut-related OSCCs. *TP53* had comparable mutation rates in areca nut-related and 42 nonareca nutrelated OSCCs (73% each), but mutations in areca nut-related cases accumulated in the p53 binding domain. For *FAT1* and *CDKN2A*, the overall mutation rates in areca nut-related cases were significantly lower (12 vs. 25% and 6 vs. 25% for *FAT1* and *CDKN2A*, respectively), but inactivating mutations were highly clustered. All *CDKN2A* mutations in areca nut-related cases occurred at a single recurrent inactivating position, p.R58*, whereas CDKN2A mutations in nonareca nut-related samples were widely distributed [17]. Mutations in *ATG2A* and another ATG2 homolog, *ATG2B*, were found to be mutually exclusive with *CASP8* mutations [15] in both areca nut-related and nonareca nut-related cases [17].

*TP53* and *HRAS*, which were mutated in 43.3 and 13% of cases, respectively, in one series exhibited mutually exclusive mutation patterns in 30 OSCC tumors from subjects from India with different tobacco consumption habits. A similar mutually exclusive mutation pattern was observed in the TCGA HNSCC samples, with primary site annotations of “floor of the mouth,” “base of the tongue,” “lip,” and “other ill-defined sites in the lip, oral cavity, and pharynx”. *PIK3CA* was mutated at positions p.Glu545Lys and p.His1047Arg in samples from both smokers and tobacco chewers, and amplification of EGFR was observed in 25% of the samples from patients with a history of tobacco consumption. Genomic alterations were also observed in *ERBB4* and *HRAS,* which are genes that are associated with protein tyrosine kinase 6 (PTK6) signaling [18]. Both geographic and exposure-related features are displayed in Table 2.

**Table 2:** Driver genetic events in oral SCC according to geography and risk factors/environmental exposures

	Risk factors/environmental exposures	High prevalence in comparison with OSCC in general population	Novel mutated genes
Pakistan (Ref. 39)	NA	*ASNS*	*ATG2A*, *WEE1*, *DST*, and *TSC2*
South Arabian peninsula (Ref. 13)	Shammah and EBV exposures	*TP53* and *CDKNA2* mutations, *CASP8*, and *PIK3CA* (20%), *HRAS* (15%), *FAT1* and *TP63* (10% each), and amplifications in *CCND1* (15%) and *FADD (*20%)	Mutations of *NOTCH3*, *CLTCL1, CSMD3*, *CRB1*, *OSMR*, and *TRPM2*; amplification of the proto-oncogenes *FOSL1*, *RELA*, *TRAF6*, *MDM2*, *FRS2*, and *BAG1*; deletion of the tumor suppressor *SMARCC1*
Taiwan (Ref. 15)	Betel nut chewing, cigarette smoking, and alcohol	*DHRS4, RASA1*, and *SETD8*	*CENVP*, *CHUK*, and *ELAVL1*
South and southeast Asia (Ref. 17)	Areca nut	In areca nut-related patients: *TP53* mutations accumulate on the p53 binding domain; *FAT1* and *CDKN2A* mutation rates are significantly lower; all *CDKN2A* mutations occurred at a single recurrent inactivating position p.R58*	*ATG2A, WEE1, DST*, and *TSC2*

## Neoplastic progression

*BRCA1*, *BRCA2*, and other DSB repair (FA)/BRCA pathway genes were prominent contributors to a 15-gene signature capable of discriminating progressive from nonprogressive oral leukoplakia (OLK). This was the first report of FA/BRCA (DSB) pathway involvement in the malignant transformation of OLK to oral squamous cell carcinoma [41].

The pathological mutations identified in a set of HNSCC and potentially premalignant (oral) lesion-derived cell lines and primary cells derived from potentially premalignant (oral) lesions predominantly involved a relatively small set of genes reported previously (*TP53*, *KMT2D*, *CDKN2A*, *PIK3CA*, *NOTCH1,* and *FAT1*) but also other predicted cancer drivers (*MGA*, *PABPC3*, *NR4A2*, *NCOR1,* and *MACF1*). Notably, all these mutations arise early and are present in potentially premalignant lesions. Deletions of FHIT (3p14.1) and *CSMD1* (8p23.2) were found among the earliest somatic copy number alterations. *CSMD1* deletions or promoter hypermethylation were present in all the immortalized cells from potentially premalignant (oral) lesions and occurred at high frequency in immortalized HNSCC cell lines. A significant increase in the frequency of somatic copy number alterations was observed in the known cancer drivers *NOTCH1*, *PPP6C*, *RAC1*, *EIF4G1*, and *PIK3CA* in the transition from potentially premalignant (oral) lesions to HNSCC, which was correlated with their expression. In the later stages of progression, HNSCC patients with and without nodal metastases presented some clear differences, including high copy number gains of *CCND1*, hsa-miR-548k, and *TP63* in patients with metastasis [42]. The cell line approach must be carefully considered, as the harvesting process could select those cells with particular genetic alterations. The number of mutations is greater in cell lines, as described in cell lines derived from laryngeal SCCs, which presented more mutations in the FAT and NOTCH gene families than primary tumors [43].

*TP53*, *CELSR1*, *CASP8*, and *KMT2D* are involved in OSCC transformation from oral lichen planus. In one study, these genes were mutated in 71% of 17 OSCCs and in 56% of nine transforming oral lichen planus cases, but they were not observed in 17 nontransforming oral lichen planus cases. Other known OSCC mutations were identified (*TRRAP*, *OBSCN*, and *LRP2*), but previously unreported mutations (*TENM3* and *ASH1L*) were also identified in lichen planus-associated OSCCs [44].

## Tumor mutational burden

The estimated tumor mutational burden (TMB) seems similar in different regions of the world. However, the TMB is not homogeneous among individual tumors [45]. Chalmers et al. reported that the median TMB of a series of 1184 HNSCCs was 5 mutations per Mb, but 10.1% of patients had more than 20 mutations/Mb (95% *CI* 8.5–11.9), with a maximum of 95.8 mutations/Mb. For nasopharyngeal and paranasal sinus SCCs, the median TMB was 4.5 mutations/Mb, and 9% of patients had more than 20 mutations/Mb (95% *CI* 4.4–18.2), with a maximum of 48.6 mutations/Mb. Some HNSCCs have a TMB much greater than the average value. In contrast with other tumors, this higher TMB is not related to microsatellite instability [46]. A high TMB results in an increase in the number of different neoantigens and their levels presented by tumor cells. These tumors would benefit from the administration of immune checkpoint inhibitors [47].

## Mutational signatures

Mutational signature refers to the most frequently mutated nucleotides and their relationship with neighboring nucleotides. Despite a greater number of mutations among smokers, the mutational spectrum was not enriched with G:C > T:A transversions according to the observations of Agrawal et al. [1], but others showed a mutational profile consistent with tobacco exposure. Among patients who reported a smoking history, those with the highest fraction of G-to-T transversions tended to have increased overall mutation rates. Thus, the G-to-T transversion frequency may represent a robust readout of “functional” tobacco exposure. Differences in mutation rates and G-to-T transversion frequencies by tumor site were observed even when the analysis was restricted to HPV-negative tumors. HPV-negative laryngeal cancers presented higher mutation rates and G to T transversion frequencies than HPV-negative cancers in the oral cavity, oropharynx, hypopharynx, or sinonasal cavity [2]. Similar results were reported among smokers with oral and gingivo-buccal SCCs [11].

Several mutational signatures have been validated in HNSCC: signature 4 from the COSMIC database is the most frequent signature observed among smoking-related tumors, such as lung and laryngeal squamous cell carcinomas. This smoking-related signature is characterized by C > A transversions with no preference for the trinucleotide context and is the most frequent among cases published by Stransky et al. [48, 49]. The excess of C-to-A transversions at the CCA, CCC, or CCT triplets occur in a similar fashion to those that occur after benzo[α]pyrene experimental cell exposure. Benzo[α]pyrene is the most important tobacco smoke carcinogen, which indicates the contribution of tobacco smoking to HN (and particularly laryngeal) SCC. Tumors in other HN sites, such as those in the oral cavity and pharynx, present different mutational signatures. However, signature 4 is more common among smokers with tumors at these sites. Laryngeal SCCs were found to be more strongly related to tobacco smoking and signature 4 than oral and pharyngeal SCCs. This finding supports the main role of tobacco smoking in laryngeal SCC, whereas its contribution to tumors in other HN sites is combined with that of other carcinogenic drivers [50]. A greater abundance of signature 4 mutations was confirmed for the TCGA-HNSC and HIPO-HNC cohorts, but as laryngeal cancers represent a minor subgroup within both cohorts, signature 4 was not ranked as one of the top five signatures [51]. Enrichment of COSMIC signature 29 was observed in 40% of OSCC samples from tobacco chewers [18].

Kataegis is a phenomenon of regional hypermutation in cancer genomes characterized by TC > TT and TC > TG clustered within genomic regions of several megabases with a special strand preference. The C > T mutations in the TpC dinucleotide context related to the kataegis signature have been associated with the AID/APOBEC-mediated DNA repair system as signature 2 described by Alexandrov et al. [48, 49]. A predominant mutation signature associated with cytidine deaminase APOBEC, which is correlated with the upregulation of *APOBEC3A* expression in the *APOBEC3* gene cluster at 22q13, was found in a cohort of 50 Taiwanese patients otherwise characteristic of OSCC regarding frequently mutated genes [15]. Compared with HPV-negative tumors, HPV-positive tumors are significantly associated with immune signature-related genes [5]. APOBEC3-associated TCW (TCA or TCT) motif mutations, but not total single-nucleotide variant burden, are significantly associated with inflammation, but this association is restricted to HPV-negative HNSCC samples. An APOBEC-enriched, HPV-negative subgroup presented increased T-cell inflammation and immune checkpoint expression. This APOBEC-enriched subgroup of HPV-negative HNSCC patients with a distinct immunogenic phenotype represents a candidate group for immune checkpoint inhibitor therapy [52]. Signatures associated with mutational processes of endogenous origins, including SBS2 and SBS13, associated with APOBEC cytidine deaminase DNA-editing activity were found in cell lines and patient-derived xenografts from HNSCCs [53].

Age-related signatures (signatures 1, 1B, or non-CGI) characterized by C > T transversions in CG dinucleotides in non-CPG island (CGI) regions are common in different cancer types, including HNSCC [14, 48, 49, 51]. In 120 Taiwanese patients with OSCC, signature 1 contributes to MSC1, which is associated with a high mutational rate and *ELAVL1* mutations [14].

A three-tiered model for HN carcinogenesis has been proposed based on these results. Many HNSCCs are directly linked to tobacco exposure. These patients frequently present with *TP53* and *CDKN2A* alterations, show high copy number alterations, and are HPV-negative. In contrast, HPV-related tumors have fewer copy number alterations, fewer genetic alterations (*TP53* and *CDKN2A* products are either inactivated or functionally silenced by the E6 and E7 viral genes) and a high rate of *PIK3CA* mutations. A third tier is composed of HPV-negative tumors with low copy number variations and *HRAS* mutations [54].

Signature 5 from the COSMIC database was also common among laryngeal, oral cavity, and pharyngeal cancers and was associated with smoking. In particular, this signature was correlated with pack-years in patients with pharyngeal and OC SCCs [50]. Signature 5 contributes to two of the five types of MSCs described in Taiwanese OSCCs, MSC2, and MSC3, both of which are related to tobacco smoking. MSC2 was associated with a low mutational rate and *FAT1* and *CASP8* mutations in younger patients at an advanced disease stage, nodal metastases, and tobacco smoking. MSC3 is associated with smoking in patients at an early disease stage [14].

Signature 6, characterized by CG-to-TA mutations at NpCpG and concomitant inactivation of MMR genes, has been observed in oral carcinomas [14]. Areca nut-related OSCCs are characterized specifically by the genomic signature of mismatch repair deficiency (dMMR), which could also predict the prognosis of patients with areca nut-related OSCC. In addition, elevated PD-L1 expression was also observed in both areca nut-related cases and those with a high dMMR. Further differential expression analysis and in vitro experiments confirmed the role of dMMR in the development of OSCC induced by areca nut exposure [17]. Signature 15, associated with defective DNA mismatch repair, was enriched in 80% of 30 OSCCs in one study [18].

Signature 7 is associated with UV light exposure and is particularly common in cutaneous melanomas, but it is also particularly common in oral carcinomas, possibly due to the inclusion of lip carcinomas [14, 49].

Primary laryngeal tumors had the highest mutational count in the HIPO-HNC cohort. Unsupervised hierarchical clustering identified four clusters (A, B, C1, and C2) according to the relative distributions of signatures 1, 2, 3, 13, and 16, the most abundant mutational signatures in the TCGAN-HNSC cohort. Cluster A had APOBEC-related signatures 2 and 13, Cluster B had signature 3, and Cluster C had high relative contributions from signatures 1 and 16 [51]. Interestingly, signature 16, which was originally related to liver cancer [49, 50], was later validated in a subset of HNSCCs [55].

## Prognosis

Oral cavity tumors with favorable outcomes show infrequent copy number alterations in conjunction with activating mutations in *PIK3CA* or *HRAS*, coupled with inactivating mutations in *CASP8, NOTCH1,* and *TP53* [4]*. CDKN2A* mutations or deletions, *TP53* mutations, *CCND1* and *FGFR1* amplifications, alterations in the cell cycle and *PIK3/AKT/MTOR* pathways and more than 3 genomic alterations negatively impact the overall survival of HNSCC patients [56]. In nasopharyngeal carcinoma, RAS/*PI3K/AKT* pathway alterations result in worse overall and disease-free survival in patients with this disease [57]. Mutations in *TP53* are significantly associated with poorer overall survival in patients with oral cancer [58]. Hotspot *TP53* missense mutations in HPV-negative tumors are correlated with an increased risk of locoregional recurrence compared with *TP53* wild-type tumors, and when *TP53* missense mutations co-occur with *CDKN2A* mutations, this risk is increased [9]. Mutations in *NOTCH1*, *CDKN2A*, and *TP53* are significantly associated with poorer overall survival [5].

Patients with at least one mutation were reported to have shorter disease-free and overall survival. The presence of at least one cancer-specific mutation was found to be positively associated with extensive desmoplastic stroma and an aggressive type of invasive front and was associated with the degree of differentiation [6].

Somatic frameshift events in tumor suppressor genes and increased TMB among virus-negative HNSCCs predict an anti-PD-1/L1 response [59]. TMB and clonality-weighted TMB were also significantly associated with objective response in a series of recurrent or metastatic HNSCCs treated with pembrolizumab, which suggests that TMB and PD-L1 may represent distinct and complementary biomarkers that predict the response to anti-programmed death therapies in HNSCC patients [60].

*PIK3CA* amplification (but not *PIK3CA* mutation) was associated with poorer progression-free survival (PFS), while *RAS* mutations were associated with poorer PFS and overall survival (OS) in a series enriched in patients with oropharyngeal tumors. Eight patients with refractory HNSCC were enrolled in clinical trials matched with the MPS results. Four patients (three treated with PIK3/mTOR inhibitors and one with a novel EGFR inhibitor) achieved a partial response [8].

A nine-gene panel (*RYR1*, *HLA*-*B*, *TSHZ2*, *PCDH17*, *DNAH17*, *GRID1*, *SBNO2*, *KSR2*, and *GCN1L1*) has been shown to predict survival outcomes in patients with OSCCs, but this panel did not perform well when its prognostic value was validated using the TCGA database, particularly among female patients [61]. *NOTCH1* mutations are associated with worse overall survival in patients with OSCC [62]. Mutational signatures had a significant, negative impact on the disease-free survival and overall survival in subgroups of patients with stage III and IV OSCC. Several important signaling pathways were frequently altered in this cohort. Alterations in the NOTCH, RTK/RAS/MAPK, and TGF-beta pathways led to a significant and negative impact on disease-free survival in a specific subgroup of patients [63].

Eighty-six percent of single somatic variants (SSVs) detected in synchronous metastases can be attributed to the primary tumor, whereas only 60% of the SSVs detected in recurrent tumors can be identified in the corresponding primary tumor. *ITPR3* and *DDR2* mutations are consistently found in metastatic lymph nodes and/or recurrent tumors but are absent in the corresponding primary tumors. Tumors harboring *DDR2* mutations could be more sensitive to SFK inhibitors, as HNSCC cell lines with endogenous and engineered *DDR2* mutations are more sensitive to the SFK inhibitor dasatinib than those with wild-type *DDR2* [64].

A consistent classification of HNSCC into four groups according to gene expression patterns failed to show prognostic value. The so-called “atypical” subtype is enriched in HPV-positive tumors, the “classical” subtype expresses genotoxic damage, the “basal” subtype is predominantly in the oral cavity, and the “mesenchymal” subtype is considered to be related to epithelial‒mesenchymal transition [65]. More recently, a three-tiered classification has shown prognostic value for both SCC (including cervix, HNSCC, esophageal, and lung cancer) and HNSCC. Cluster 1, characteristic of cervical SCC and a subset of HNSCCs, presents the characteristic mutational pattern of HPV-positive tumors and the best patient outcome, whereas Cluster 3, with the highest number of cases with *TP53* or *CDKN2A* mutations, resembles the “classical” subtype associated with genotoxic damage. A substantial subset of 514 HNSCCs clustered with either cervical (CSCCs) or lung (LSCCs) SCCs, which suggests that these tumors share more characteristics with tumors of other origins than with other HNSCCs. Moreover, the evolution of these tumors was similar to that of the clustered tumors: 61 subtype 1 tumors behaved more like CSCCs than like other HNSCCs, and the same was true for the 70 subtype 3 tumors, which had the worst prognosis. The mutational analysis was restricted to the 21 genes most frequently mutated in HNSCC. *TP53* alterations (mostly missense mutations) were more common among subtypes 2 (43%) and 3 (53%). *CDKN2A* was the most frequently deleted gene in subtypes 2 (48% of alterations, mostly deletions) and 3 (53% of alterations, mostly mutations) but was rarely deleted in subtype 1 (8%, all deletions). *NOTCH1* was most frequently altered in subtype 2 (16% vs. 5% in subtype 1 and 7% in subtype 3). *PIK3CA* was frequently altered throughout the three subtypes but had the highest prevalence among subtypes 1 (36%) and 3 (53%). Subtype 3 had frequent mutations in *AJUBA, MUC17, KMTD2,* and *NFE2L2* in contrast with other types/subtypes, which indicates that although subtype 3 HNSCC shares expression profiles with LSCC, the underlying mechanisms differ [66]. Distinct subgroups containing loss-of-function alterations in the chromatin modifier *NSD1* and in the WNT pathway genes *AJUBA* and *FAT1* and that feature activation of the oxidative stress factor *NFE2L2*, occur mainly in laryngeal tumors [4].

According to data from the TCGAN database, mutant allele tumor heterogeneity (MATH) was associated with worse overall survival in HNSCC patients, including those with oral, oropharyngeal, and laryngeal tumors. High MATH was also associated with HPV-negative HNSCCs. In the same series, HPV positivity and high estrogen receptor α expression conferred a favorable prognosis. The combination of the three items conferred the best discriminant prognosis [67].

## Actionability

Many genetic alterations could be useful markers for therapeutic decision-making. Three-quarters of patients with laryngeal and hypopharyngeal cancers harbor candidate actionable or prognostic alterations in genes belonging to the RTK/ERK/PI3K, cell cycle, and DNA damage repair pathways [35]. Loss-of-function mutations in *STK11* (LKB1) result in the activation of mTORC1 signaling and can sensitize cells to mTOR inhibition [21]. Targetable genomic alterations were identified in *FGFR1*, *DDR2*, *EGFR*, *FGFR2*/*3*, *EPHA2*, and *PIK3CA* [7]. By defining the catalog of targetable genomic alterations in a series of 120 OSCCs in male Taiwanese patients, 58% of the tumors were found to carry at least one aberrant alteration potentially targeted by US Food and Drug Administration (FDA)-approved agents. Strikingly, when the p53-cell cycle pathway (*TP53* and *CCND1*) is targeted by drugs investigated in phase I‒III clinical trials, tumors with possible actionable alterations are predominantly located in the tongue, which suggests a better prediction of sensitivity to current targeted therapies [14]. However, both the number of cases with putatively actionable genetic alterations and the level of evidence of actionability are low: 50% or more of the samples tested in one study had no actionable alterations, and most had only one or two. Moreover, the level of evidence of actionable alterations is still investigational or hypothetical [68]. In the paper by Bailey et al., 71% of 492 HNSCC patients had at least one putatively actionable SNV/indel/CNV, but only 16% of 502 patients had a druggable mutation included in the Database of Evidence for Precision Oncology, and of those mutations, most are limited to preclinical evidence [31].

The expression of a constitutively active mutant *MAP2K1* (p.K57E) resulting in alterations in the MAPK pathway was observed in erlotinib-resistant SCC-R cells. These findings suggest that targeting the MAPK pathway could be a strategy for the treatment of erlotinib-resistant HNSCC [69].

In one series, *FAT1* mutations were highly enriched in cisplatin responders, and potentially targetable alterations, such as *PIK3CA* E545K and *CDKN2A* R58X, were noted in 14 patients (15%) [5]. Alterations in clinically actionable targets, including *ERBB4*, *HRAS*, *EGFR*, *NOTCH1*, *NOTCH4*, and *NOTCH3,* were also observed. *NOTCH1* was mutated in 33% of the samples and harbored both truncating and missense variants. Copy number alterations were observed in 67% of the 33 OSCC samples. Several genes associated with nonreceptor tyrosine kinase signaling are affected in OSCC, and thus these molecules can serve as potential candidates for therapeutic targeting in OSCC [67]. Cisplatin resistance and the development of distant metastases are associated with dysregulation and epigenetic reprogramming of the KEAP1–NRF2 signaling pathway [70, 71].

When whole-genome and transcriptomic sequencing data of OSCC patient samples from 26 individuals under 50 years of age were compared with the data of OSCC patients over 50 years of age (*n* = 11) from the TCGA, a molecular signature comprising *EGFR* amplification and increased EGFR RNA abundance specific to the subset of young OSCC patients was identified for the first time. Furthermore, through functional assays using patient tumor-derived cell lines, *EGFR* amplification resulted in increased activity of the EGFR pathway. An EGFR-amplified patient-derived cell line was responsive to EGFR inhibition by clinically relevant EGFR inhibitors, which suggests that *EGFR* amplification represents a valid therapeutic target in this subset of OSCC patients [19].

## Summary and conclusions

In summary, MPSTs have revealed a complex molecular landscape in HNSCC, as many different driver genes are present in small fractions of different tumors, which enhances the effect of more commonly involved genes, such as *TP53* and *CDKN2A*. MPSTs have highlighted the role of previously unreported or underrepresented genes, such as *PIK3CA*, NOTCH family genes, *FBXW7*, and many others to a lesser extent. *NOTCH1* mutations were described for the first time in HNSCC and revealed a tumor suppressive behavior of *NOTCH1* in HNSCC, which contrasts with the common oncogenic effect of *NOTCH1* mutations in hematolymphoid malignancies. Recent reports have described *NOTCH1* mutations with a predictive oncogenic effect, which adds complexity to the mutational profile of HNSCC. Different populations have different mutational profiles, which indicates the role played by various environmental and genetic factors. *PIK3CA* is the most frequently mutated oncogene, and the PI3K pathway is the most commonly activated oncogenic pathway in HNSCC. In addition, *PIK3CA* and *TP53* mutations are distributed in a mutually exclusive pattern.

Mutational signature analysis revealed different mechanisms at different head and neck sites, with laryngeal tumors showing the highest prevalence of smoking-related signatures. However, all sites showed an association between smoking exposure and smoking-related signatures.

Some gene mutations or pathway alterations have prognostic value or are correlated with tumor evolution. Unfortunately, the number of actual targetable alterations with direct impacts on patient therapy remains low.

## Abbreviations

*AID*: Activation-induced cytidine deamination
*APOBEC*: Apolipoprotein B mRNA editing enzyme catalytic polypeptide
*CNA*: Copy number alterations
*COSMIC*: Catalog of somatic mutations in cancer
*CSCC*: Cervical squamous cell carcinoma
*dMMR*: Defective multiple mismatch repair
*DSB*: Double-strand break
*EBV*: Epstein-Barr virus
*EGF*: Epidermal growth factor
*FA*: Fanconi anemia
*FDA*: US Food and Drug Administration
*HN*: Head and neck
*HNSCC*: Head and neck squamous cell carcinoma
*HPV *: Human papilloma virus
*LOH*: Loss of heterozygosity
*LSCC*: Lung squamous cell carcinoma
*MATH*: Mutant allele tumor heterogeneity
*MMR*: Multiple mismatch repair
*MPST*: Massive parallel sequencing techniques
*MSC*: mutational signature cluster
*NF-κB*: nuclear factor κB
*OC*: oral cavity
*OLK*: oral leukoplakia
*OP*: oro-pharyngeal
*OSC*: Oral squamous cell carcinoma
*PEST*: proline, glutamate, serine, and threonine
*PI3K*: phosphatidyInositol-4, 5-biphosphonate 3-kinase
*SCC*: squamous cell carcinoma
*SSV*: single somatic variant
*TCGA*: The Cancer Genome Atlas
*TCGAN*: The Cancer Genome Atlas Network
*TMB*: tumor mutation burden
*UV*: ultraviolet
*WES*: whole exome sequencing
*WGS*: whole genome sequencing
